# Participatory action research to develop and implement multicomponent, multilevel strategies for implementing colorectal cancer screening interventions in American Indian communities in New Mexico

**DOI:** 10.1186/s43058-024-00591-y

**Published:** 2024-05-10

**Authors:** Prajakta Adsul, Kevin English, Cheyenne Jim, V. Shane Pankratz, Nicholas Edwardson, Judith Sheche, Joseph Rodman, Jimmie Charlie, John Pagett, Jonathan Trujillo, Jillian Grisel-Cambridge, Steven Mora, Kaitlyn L. Yepa, Shiraz I. Mishra

**Affiliations:** 1 University of New Mexico Comprehensive Cancer Center, Albuquerque, NM USA; 2 Department of Internal Medicine, School of Medicine, University of New Mexico, Albuquerque, NM USA; 3 Albuquerque Area Southwest Tribal Epidemiology Center, Albuquerque, NM USA; 4 University of New Mexico School of Public Administration, Albuquerque, NM USA; 5 Kewa Pueblo Health Corporation, Kewa Pueblo, NM USA; 6 Jemez Health & Human Services, Jemez Pueblo, NM USA; 7 Department of Pediatrics, University of New Mexico Health Sciences Center, 1 University of New Mexico, MSC 10 5590, Albuquerque, NM 87131 USA; 8 Department of Family and Community Medicine, University of New Mexico Health Sciences Center, 1 University of New Mexico, MSC 10 5590, Albuquerque, NM 87131 USA

**Keywords:** Implementation science, Colorectal cancer, Cancer screening, American Indian, Tribal communities, Community based participatory research, Implementation strategies

## Abstract

**Background:**

Despite the effectiveness of colorectal cancer (CRC) screening, American Indians (AIs) have low screening rates in the US. Many AIs receive care at Indian Health Services, Tribal, and Urban Indian (I/T/U) healthcare facilities, where published evidence regarding the implementation of CRC screening interventions is lacking. To address this gap, the University of New Mexico Comprehensive Cancer Center and the Albuquerque Area Southwest Tribal Epidemiology Center collaborated with two tribally-operated healthcare facilities in New Mexico with the goal of improving CRC screening rates among New Mexico’s AI communities.

**Methods:**

Guided by the principles of Community Based Participatory Research, we engaged providers from the two tribal healthcare facilities and tribal community members through focus group (two focus groups with providers (*n* = 15) and four focus group and listening sessions with community members (*n* = 65)), to elicit perspectives on the feasibility and appropriateness of implementing The Guide to Community Preventive Services (The Community Guide) recommended evidence-based interventions (EBIs) and strategies for increasing CRC screening. Within each tribal healthcare facility, we engaged a Multisector Action Team (MAT) that participated in an implementation survey to document the extent to which their healthcare facilities were implementing EBIs and strategies, and an organizational readiness survey that queried whether their healthcare facilities could implement additional strategies to improve uptake of CRC screening.

**Results:**

The Community Guide recommended EBIs and strategies that received the most support as feasible and appropriate from community members included: one-on-one education from providers, reminders, small media, and interventions that reduced structural barriers. From the providers’ perspective, feasible and acceptable strategies included one-on-one education, patient and provider reminders, and provider assessment and feedback. Universally, providers mentioned the need for patient navigators who could provide culturally appropriate education about CRC and assist with transportation, and improved support for coordinating clinical follow-up after screening. The readiness survey highlighted overall readiness of the tribal facility, while the implementation survey highlighted that few strategies were being implemented.

**Conclusions:**

Findings from this study contribute to the limited literature around implementation research at tribal healthcare facilities and informed the selection of specific implementation strategies to promote the uptake of CRC screening in AI communities.

**Supplementary Information:**

The online version contains supplementary material available at 10.1186/s43058-024-00591-y.

Contributions to the literature

- This study adds to the very limited implementation research specific to healthcare facilities that are operated by the Tribal communities
- Study findings highlight the role of community-based research approaches and the use of facilitation to improve the uptake of evidence-based colorectal cancer screening in Tribal communities
- Findings from this study inform the ongoing implementation assessments specific to colorectal cancer screening in Tribal communities

## Background

Colorectal cancer (CRC) is the third most frequently diagnosed cancer and the third leading cause of cancer death among men and women in the United States (US). Significant racial disparities persist in CRC incidence and mortality [1]. The 2023 update to the colorectal cancer statistics shows that among the five racial and ethnic groups, American Indian/Alaska Native (AI/AN) individuals and Black individuals have the highest CRC incidence and mortality [2]. Since 2014, AI/ANs have experienced either no change or an increase in CRC incidence [3–7], disproportionate diagnosis of late-stage disease [3, 7–9], and poorer overall [10, 11] and CRC-specific [9, 10] 5-year survival rates.

Several screening tests (i.e., stool-based tests, flexible sigmoidoscopy, computed tomography colonography, flexible sigmoidoscopy, and colonoscopy) show evidence in reducing CRC-associated mortality [12]. In 2021, the United States Preventive Services Task Force (USPSTF) recommended screening for all adults between the ages of 45–75 years with any of these screening tests at appropriate time intervals [13]. Low rates of CRC screening, however, present an urgent public health concern in the US. The 2020 data from the Behavioral Risk Factor Surveillance System suggest lower rates of adults being up-to-date with the USPSTF recommendations in New Mexico (NM) (68.8%) compared to the overall US population (74.2%) [14]. Screening rates for AI/AN populations however, remain low [7, 15, 16]. Based on Indian Health Service (IHS) Government Performance and Results Act (GPRA) FY 2021 data [17], screening rates are currently 29% for AI/ANs in the IHS Albuquerque Area. These rates only capture the active users of IHS, Tribal, and Urban Indian (I/T/U) healthcare facilities. Population-based rates, particularly in more rural areas, are undoubtedly lower than those reported above.

Even with a robust evidence-base, there is limited information on how and under what conditions the USPSTF recommendations could be implemented to improve screening uptake, reduce disparities, and reduce the CRC burden [12]. The science of implementation is well-suited to bridge such a gap between research and practice by building a knowledge base about the specific strategies that help with the adoption and integration of interventions into routine practice at healthcare settings so as to benefit population health [18]. From a public health practice perspective, The Community Guide (put forth by the Community Preventive Services Task Force) provides an important collection of evidence-based interventions (EBIs) and strategies that have been effective in previous research studies [19]. For CRC, The Community Guide recommends the use of multicomponent approaches (selected from 11 distinct approaches that include, for example, group education, reducing structural barriers, provider reminders, among others) to increase screening uptake by: (1) increasing community demand, (2) increasing community access, and (3) increasing provider delivery; each of which impacts different socioecological levels [20, 21].

Although the Community Guide recommendations (Appendix 1) provide an important first step, there is a lack of specificity around the operationalization of the EBIs and strategies in practice. For example, depending on the implementation setting (i.e., clinical or community setting), there could be multiple combinations of EBIs and strategies employed based on feasibility and appropriateness for the populations being served. To be specific, how a strategy of “provider reminders” gets implemented in a healthcare setting could range from a flag in the Electronic Health Record (EHR) system that requires support from the Information Technology (IT) services, to something that a physician assistant may ask a patient before they are seen by the physician. Each of these strategies require implementation efforts that are unique to the context of the setting and populations they serve. Furthermore, there is recognition in the field that context is dynamic and if we are to ensure sustainability of outcomes, we must follow a pathway that includes learning, optimization, and implementation of interventions [22, 23].

Tribal communities face structural challenges in securing sufficient health care resources, including those specific to addressing CRC disparities. In the IHS, per capita health care expenditures for patient health services are low, which can lead to fragmented service delivery [24]. As an example, most I/T/U healthcare facilities do not offer colonoscopies [25]. This might explain missed appointments for colonoscopies further afield; which often are due to lack of access to transportation [26, 27], familiarity with health care settings and trust with providers [28–31]. With limited resources, acute care services often take precedence over preventive health services [32]. Likewise, resource limitations can restrict staffing levels at many I/T/U healthcare facilities leading to higher provider turnover, which may result in abbreviated patient-provider encounters and insufficient communications [32]. Additionally, AI/AN populations also experience and note fear, stigma, embarrassment, privacy concerns, and strong cultural beliefs about cancer and screening services, that require careful implementation considerations [16, 28, 29, 33–35]. In these settings, cost and economic measurements are key to understanding the implementation and sustainability that are often missing from intervention studies [36]. These challenges underscore the need to implement effective multilevel and multicomponent CRC screening interventions, informed by community input that address the needs of AI/ANs seeking care at I/T/U healthcare facilities.

The National Cancer Institute (NCI) continues to invest through the Cancer Moonshot^SM^ to study the implementation of CRC screening in healthcare facilities across the US participating through the Accelerating Colorectal Cancer Screening through Implementation Science (ACCSIS) Initiative. Eight research projects, including the NM research project (one of the three research projects in the AI CRC Screening Consortium), participate in this initiative to plan, implement, and assess multicomponent, multilevel strategies to promote the uptake of CRC screening in healthcare facilities that often provide care to underserved, and racially/ethnically minoritized communities across the US.

This paper describes the design and implementation of the NM research project that incorporates community input and engagement processes, guided by the principles of Community-Based Participatory Research (CBPR) [37, 38]. These principles include: 1) an emphasis on co-learning, capacity building, and reciprocal transfer of expertise between all academic, AI/AN community, and I/T/U healthcare providers; 2) shared decision-making with respect to the project by all partners; 3) mutual ownership of the processes and products of the research enterprise by all partners; 4) a commitment to build on the strengths and resources within the community; 5) a commitment for balance between research and action; 6) emphasis on problems of local significance in an ecological context; 7) widespread dissemination while recognizing the privacy and confidentiality of Tribal participants, and 8) a commitment to sustainability.

The study team is composed of researchers from the University of New Mexico Comprehensive Cancer Center (UNMCCC) and the Albuquerque Area Southwest Tribal Epidemiology Center (AASTEC), a program of the Albuquerque Area Indian Health Board, Inc. that provides leadership, technical assistance, training, and resources to the 27 AI/AN Tribes, Bands, Pueblos, and Nations within the IHS Albuquerque Area. This study builds upon prior successful research partnerships among all entities and takes place in three phases: Planning (Year 1), Pilot (Year 2) and Implementation (Years 3–5). In particular, this paper describes activities undertaken in the Planning and Pilot Phases (Years 1–2) of the project, with formative research activities centered upon environmental scans using multiple methods (i.e., focus groups and quantitative assessments) with Tribal members and staff of the healthcare facilities in two Tribes to ensure that our study builds upon existing best practices and fits the community, cultural, and healthcare facility context, within each Tribe.

## Methods

### Research setting and key partners

Two Tribal communities (deidentified; referred to here as Tribe 1 and Tribe 2) participated in research activities during the Planning and Pilot Phases of the study. Both Tribes, located in rural NM, operate their own healthcare facilities, and have baseline CRC screening rates slightly below the regional average of 29%. The study received approvals from the University of New Mexico (UNM) Health Sciences Center Human Research Review Committee [18–636] and the Southwest Tribal Institutional Review Board (protocol SWT-2018-005). This study was conducted as part of the NCI-funded ACCSIS Program consortium. The overall aim of ACCSIS is to conduct multi-site, coordinated, transdisciplinary research to evaluate and improve colorectal cancer screening processes using implementation science.

### Study design and analyses

During the Planning and Pilot Phases, the research team connected with individuals from healthcare facilities in the two Tribes, using existing working relationships. In each tribally-operated healthcare facility, the research team facilitated the establishment and mobilization of a Multisector Action Team (MAT). Tribe 1’s MAT designated co-champions and representatives from 11 relevant sectors including: health administration, physicians, tribally-operated healthcare facility nursing, public health nursing, community health workers, medical records, purchase and referred care, medical assistants, quality assurance, patient registration, and EHR. Tribe 2’s MAT also designated a champion and included representatives from 14 different sectors including: health administration, physicians, tribally-operated healthcare facility nursing, public health nursing, community health workers, medical records, purchase and referred care, medical assistants, quality assurance, EHR, transportation, pharmacy, patient registration and behavioral health. The research team provided the MAT with current USPSTF CRC screening guidelines and discussed The Community Guide’s recommendations for EBIs and strategies for increasing CRC screening uptake. Through multiple monthly meetings, research team members facilitated discussion among MAT members to help them select, prioritize, and implement a comprehensive set of activities to address contextual barriers and improve the delivery of CRC screening at their tribally-operated healthcare facilities.

Broadly, these research activities were formative in nature and aimed at understanding the implementation context in the healthcare facilities, while incorporating the perspectives of the Tribal members that sought and received care at these facilities. At each Tribe/tribally-operated healthcare facility, we conducted focus group discussions and listening sessions with Tribal members to understand the social, cultural, and economic factors that influence CRC screening uptake in their community. Across the two tribes, a total of 65 Tribal members participated in three group discussions and two listening sessions that took place in February and March 2019. The discussion guide used with Tribal members is provided in Appendix 2, and included questions around: screening processes, education and outreach, and personal experiences. We also conducted focus group discussions with the MAT members. A total of 15 individuals participated in two focus groups that were conducted in February 2019. The discussion guide for MAT members is also provided in Appendix 2. These questions were similar to those for the Tribal members in eliciting the overall perceptions towards strategies that could be implemented within their healthcare facilities.

Five focus group discussions and listening sessions were audio recorded and transcribed, while the sixth group discussion, a listening session, was limited to meeting notes taken by the facilitator. The transcripts were analyzed using a content analysis approach [39]. Both the facilitators and the analysts were trained with a master’s in public health, with at least one individual in each group with a doctorate. All facilitators underwent trainings to prepare for the focus groups and discussion sessions, with a guide that was created with input from all research team members. For the analysis, we also provided trainings for the analysts in coding, grouping, and summarizing themes for the analysis. Two team members (PA, JR) coded each of these transcripts using the Dedoose software [40], independently to identify overarching categories and concepts, loosely based around the recommendations from The Community Guide. They met to discuss these concepts and ultimately developed a codebook that operationalized each code. They also resolved disagreements about the codes (for instance, about the operationalization of the codes or applicability of specific codes to specific excerpts), modified codes as necessary, and finalized coding through discussion and consensus. A third team member (SM) resolved any discrepancies in the coding process.

To further inform implementation, we conducted two surveys with MAT members. The first survey was an implementation survey, that documented the extent to which their healthcare facilities were implementing EBIs and strategies as recommended by The Community Guide [20] to understand what strategies were currently being implemented or being considered for future efforts. Adapted from a previous research study where the survey underwent rigorous development and pilot testing [41], this survey helped query whether the healthcare facilities could implement additional strategies to improve uptake of CRC screening. The second survey was the Organizational Readiness survey, which has been previously validated as the Organizational Readiness for Implementing Change (ORIC) measure [42]. Both surveys were administered during a MAT meeting with the research team. All available MAT members from the two healthcare facilities participated in answering the survey questions using a group consensus-based approach, which has been supported in previous studies [43, 44]. Briefly, facilitators read out the survey items during a meeting, the group provided some answers, and in case of discrepancies there was a facilitated discussion on the answers. At the end of the discussion, the facilitators then queried the group to ensure agreement on the final answer. A total of five and eight MAT members from Tribe 1 and Tribe 2, respectively, participated in the survey. The information from these surveys and the focus groups discussions informed the MAT’s identification and selection of appropriate, feasible, and acceptable strategies for implementation at the healthcare facilities.

## Results

### Characteristics of the focus group participants and the MAT members from each facility

Tables 1 and 2 describe the characteristics of the focus group participants and the MAT members. Findings from the focus group discussion with community members and providers are organized in themes below and Table 3 presents representative quotes from community members and providers cross-referenced with the Community Guide recommended EBIs.

**Table 1:** Characteristics of the individuals involved in the focus groups and surveys

	**Focus groups**	
	**Community members**^**a**^** (*n* = 21)**	**Healthcare Providers (*n* = 15)**
**Age**		
20–30 years	0 (0%)	1 (7%)
31- 40 years	0 (0%)	1 (7%)
41–50 years	0 (0%)	7 (47%)
51- 60 years	9 (47%)	2 (13%)
60 years and above	10 (53%)	4 (27%)
Missing	2	0
**Gender**		
Male	10 (50%)	2 (13%)
Female	10 (50%)	13 (87%)
**Race**		
White	0 (0%)	7 (47%)
American Indian or Alaska Native	18 (100%)	7 (47%)
Black	0 (0%)	0 (0%)
Don’t know/Not sure	0 (0%)	1 (7%)
Missing	3	0
**Ethnicity**		
Hispanic, Latino, or of Spanish origin	1 (5%)	1 (7%)
Not Hispanic, Latino, or of Spanish origin	19 (95%)	14 (93%)
Missing	1	0
**Education**		
Less than high school	1 (6%)	0 (0%)
High school or equivalent	10 (56%)	0(0%)
Some college, no degree	4 (22%)	1 (7%)
Associate, Baccalaureate, or Masters’	3 (17%)	9 (60%)
Doctorate or Professional	0 (0%)	5 (33%)
Missing	3	0

^a^No demographic data were collected at the listening sessions

**Table 2:** Characteristics of the individuals involved in the surveys

	**Surveys with Multisector Action Team members**	
	**Tribe 1 (*n* = 5)**	**Tribe 2 (*n* = 8)**
**Age**		
20–30 years	0 (0%)	0 (0%)
31- 40 years	2 (40%)	1 (14%)
41–50 years	2 (40%)	4 (57%)
51- 60 years	1 (20%)	1 (14%)
60 years and above	0 (0%)	1 (14%)
Missing	0	1
**Gender**		
Male	0 (0%)	1 (13%)
Female	5 (100%)	7 (88%)
**Race**		
White	1 (20%)	2 (25%)
American Indian or Alaska Native	3 (60%)	6 (75%)
Black	1 (20%)	0 (0%)
Don’t know/Not sure	0 (0%)	0 (0%)
**Ethnicity**		
Hispanic, Latino, or of Spanish origin	0 (0%)	0 (0%)
Not Hispanic, Latino, or of Spanish origin	5 (100%)	8 (100%)
**Education**		
Less than high school	0 (0%)	0 (0%)
High school or equivalent	0 (0%)	0 (0%)
Some college, no degree	1 (20%)	1 (13%)
Associate, Baccalaureate, or Masters’	4 (80%)	6 (75%)
Doctorate or Professional	0 (0%)	1 (13%)

**Table 3:** Representative quotes from focus group discussions with community members and providers for the community guide recommendations

The Community Guide recommendations	Provider quotes	Community quotes
One-on-one education	P1. “I think when you talk to them you’ve got to really stress prevention, early detection. Maybe you mention it. A screening for cancer, but just really stress it a little bit. ‘This is a preventative thing.’” (Tribe 2) P2. “I just provide information. I feel like I’m there to educate them, and it doesn’t matter what culture they come from as far as educating. I feel like that’s my job and to remain flexible. That’s why I try to educate, ‘[*colonoscopy]* is the gold standard…and here are other options.’” (Tribe 1) P3. “I use myself a lot of times. I set an example. I tell them, ‘I had it done. I had to go through this procedure. Yeah, I was kind of worried in a way about what they might find, but everything came out fine.’ I just tell them about my experience and say, ‘I did it, and I’ll probably have to go again for another one.’” (Tribe 2)	C1. “Wellness, when they have the wellness program, bring it out. People want to get educated or want to know more about how you’re functioning deep inside.” (Female, Tribe 1) C2. “I believe that they need to be more tactful to where they’re not being alarmed, because you are preventing that person from coming in to have that screening done. I think that there should be a way to approach your patients to where they may be willing to come in to get that screening.” (Female, Tribe 1) C3. “I had mine done when I turned 50 right away, because the physician that I was seeing, we had a really good rapport, relationship, you know the doctor-patient relationship; but I trust this guy. He made me realize that I needed to do that, rather do it now than down the road. He’s the one that got me started, and I did it right away.” (Male, Tribe 1)
Client reminders	P4. “I think maybe reminder letters might be good, too, to say, ‘It’s time,’ like you said, ‘Congratulations! Time for you to get your annual exam. You need this and this and this. Please take this letter to your provider.’” (Tribe 2) P5. “…the reminder cards that are available to us. We can just start mailing those out like crazy.” (Tribe 2) P6. “I’m wondering if we can have—there’s a way through one of the computer programs to print out a list of people due for whatever they’re due for, but I just wonder if we can take our computer program and get all the February birthdays for everybody from 18 to 75 and just mail out a birthday card that says, ‘Happy birthday! Remember to come in for your annual exam.’” (Tribe 1)	C4. “A letter and then a phone call, reminder calls.” (Female, Tribe 1) C5. “Mail is better. We are a small community here. We get the mail, and we say, ‘Did you get this one? Did you get this?’ I said, ‘I don’t care. He’s got it.’ It spreads around, and I’m sure that’s the best way.” (Male, Tribe 1) C6. “Yeah, [mail is better than text messages] because the text is going to be in the [indecipherable] but mail, like letters, would be some other family member who would see it.” (Male, Tribe 1)
Small media	P7. “I have had people who say, ‘Yeah, I’d be willing to publish an article [in] the newsletter.’ I’ve asked people about that, but I haven’t gone any further. It’s something I’ve thought about but not done anything about.” (Tribe 1) P8. “I’d really like to see it on the TV out in the waiting area to just have some scenarios of colorectal [cancer screening].” (Tribe 2)	C7. “We have a lot of senior centers that we can provide those flyers out there, and then we’re all [inaudible] information pamphlets and information [inaudible]. That would be awesome.” (Female, Tribe 1) C8. “… when I come [to the clinic] if I look at a new brochure, I’ll take that time to read it.” (Female, Tribe 1) C9. Moderator: “Would pamphlets help?” Female: “Yeah, I think so. Tell them what they are and what the benefits are from getting those checkups.” (Female, Tribe 1) C10. “Put it in bold letters, not too much information, but direct. The letter to the client is, I think that something like the—I know you guys have it out on the newsletter, but individual sheets. It’s just direct information to all of the community members. I think those should be mailed out.” (Male, Tribe 1)
Client incentives	P9. “When we work with our *[facility name redacted]* clients, it’s an incentive-based program. When they come to any of our skill-building seminars, which we hold every month, they get an incentive for that, plus we have lunch for them, but I think I’ve seen the number of clientele coming in grow. We have a lot more people; we have 25, 30 people that come to our presentations. I’m anxious to do the one on colorectal to see if it really makes a difference in people coming back.” (Tribe 2)	Not discussed
Group education	P10. “I can envision like if you had a certain day out of the week or the month, you have a men’s wellness [clinic]. To have the education before the event, like a group education, and then they have that knowledge to say, ‘Now I know what it is to take this test.’” (Tribe 2) P11. “If people know that they’re not alone in having to go through what they’re going through, it seems to have some sort of a reassuring—a very powerful reassurance in this community that I’ve seen.” (Tribe 1)	C11. “Have more sessions with the people that are non-workers.” (Female, Tribe 1) C12. “How about talking about trainings that I go through with them when I come home and I talk to them about my family. Then, if they have friends over, if they have anybody over, then they can engage into the conversation by asking questions. That’s how I communicate my trainings with not just my family, but other people as well, because I try to educate as much as I can, because it is important.” (Male, Tribe 1) C13. “… Any health conferences that you guys have, anything. It doesn’t have to be just a certain time or a different or, like you said, for that time.” (Female, Tribe 1)
Reduce structural barriers	Not discussed	C14. “Somebody who speaks *[tribal language redacted]* too.” (Female, Tribe 1) C15. “It would still be helpful with the English and a [*tribal name redacted*] person.” (Female, Tribe 1) C16. “People have turned away themselves, because when they call in, they say, ‘You can be seen two or three days from now.’ If you’re in pain, who wants to wait two or three days?” (Female, Tribe 1) C17. “A lot of people don’t like to come on their own or make appointments. We’re not used to making appointments here.” (Female, Tribe 1) C18. “We have no more walk-in sufficiency, no more walk-ins available… they have been allowed to serve the tribal corridor, so we have non-Indians; we have anybody and everyone here. Even that’s a turn off for our people, because they are bringing in money, so of course they’re going to get the treatment. Then, these people are told, ‘Well, we have to schedule.’ I think that’s the number one problem.” (Female, Tribe 1) C19. “Why do we have to make an appointment when you’re not going to be seen at the time that they make the appointment for you? This is ridiculous. I told the lady who administers the front desk, the clinic desk, so she made an attempt to go see how busy they are back there. She comes back and says, ‘Oh, well, they’ll come to you in five to ten minutes.’ ‘No, I don’t want to wait ten more minutes. My appointment time was at this time. I should have been seen at that time. Why are you telling me to wait ten more minutes?’” (Female, Tribe 1) C20. “A person had come through the clinic that is really rare to see a doctor and prompted himself to get enough encouragement to come here to be seen, but only he turned away because they didn’t have any openings. Come back at a later date, but the later date never happened because the person said, ‘I tried, but now I’m not going to go back.’ (Male, Tribe 1) C21. “It’s been several months since he gave me that recommendation [for a colonoscopy]. I’m waiting for a follow-up, but nothing happened yet.” (Male, Tribe 1) C22. “The turnaround [for follow-up appointments] is just poor.” (Male, Tribe 1)
Provider reminders	P12. “Or even medical records could set—when patients come in for their sore throat or whatever, they say, ‘What your provider would like is for you to come in a week or two from now when you’re feeling better. Don’t come in with any complaint. Just come in so we can talk about some things that we need to do as far as your overall health.’” (Tribe 1) P13. “Currently, I think in the clinic, what’s happening is we’ve tried to focus a little bit more on—there’s a reminder box in the EHR. If we click on that, we can see that they’re due for colorectal screening. It’s just basically provider dependent and visit dependent. We could be slammed and not even hit the reminder box, or we can have a little extra time and go through everything that they’re due for. (Tribe 1) P14. “Because our system hasn’t be updated, there is no accurate prompt [for screening] at this time.” (Tribe 2) P15. “I try to routinely make sure that everyone’s up to date with stuff. We’ll encourage it. I know the lab hands out the FIT kits and so does pharmacy if they notice it as well. However, our system is outdated as to who is up to date.” (Tribe 2) P16. “When we moved to the new EHR, the old system did not filter in. You had to in manually and literally put the information from the old system. Like, in our new system, which is only a year and a half old, roughly, there may be nothing in there about whether they’ve ever had a colonoscopy or not.” (Tribe 2) P17. “Yeah, we don’t know who really needs it [CRC screening], because we click in the reminders and it says that everything needs to be done […] But we have to go through and update the data. It’s the biggest problem […] Yes, the data is very out of date […] At times, I have been recommending to take a FOBT test and the patient said, ‘I had colonoscopy done last month,’ but it’s not in there, but we need that. We need to have all of that in the system. We don’t know want to give an FOBT test to someone who had it already done, a colonoscopy.” (Tribe 2) P18. “I don’t [know] whether or not we would have any luck with having *[redacted]*’s staff handing these [FIT kits] out and then requesting that the patient makes an appointment to see a provider to bring it back, because I think somebody still has to put the order in and somebody has to be responsible for finding the result.” (Tribe 1)	C23. “I believe that the providers should have that information in the patient’s chart to where, ‘Okay. This patient is going to be turning 50, so we need to have this information in the chart to where, once she opens that chart, she needs to address the issues that this patient might be facing.’” (Female, Tribe 1) C24. “The charts, the records, they’re the ones who are taking care of the patient’s record […] files, have a sheet where they check off, ‘This person needs to have this checked.’ Inserted in the patient’s chart once they’re updating, checkup [inaudible], to where the providers need to be consistent, not just pick every other person, because right now a lot of us didn’t know. I’m just being asked by people that have been asked to come and be screened.” (Female, Tribe 1) C25. “The practitioner, or whoever their doctor is, they should have it on the screen or on a form reading it to them. That way, they can protect themselves too from being told that they never say anything like that to the patient. The patient can be told, ‘This was told to you on the day that you were seen on a certain date,’ or maybe they didn’t come in for that, but just to have the reminder out for the patient or customer that comes in.” (Female, Tribe 1) C26. “You’ve got to have a new patient form. When you go into the different hospitals or different specialists, when you do down there, they give you same form that you have to check everything. When you do have questions [inaudible]. They need a form like that […] Check sheet, that way they check them and if they say, ‘You don’t have one,’ you said, ‘Yeah, I did.’ ‘When?’ ‘I don’t know.’” (Female, Tribe 1)
Provider assessment & feedback	P19. “We should each have our report parts, so to speak, with, ‘This is what the facility is doing and this is what you’re doing,’ so we know where we are, not like, ‘This is what the facility is doing. This is what you’re doing. Each person is doing this. This is the high-producing person, and you are not the high-producing person, so you need to get onboard with this person.’” (Tribe 1) P20. “Yeah, [reports about our metrics] where’s it’s one-on-one and not comparing you to every other provider, where it’s just, ‘What is my ratio?’ She gets her ratio, and not say, ‘Out of all of you, you’re the naughty one.’ That was how they did at *[facility name redacted]”* (Tribe 1)	Not discussed
Multicomponent interventions	P21. “I think the multicomponent intervention strategy would be helpful if maybe we could find a way, like I was saying earlier, for it to be a standing order where we don’t have to—personally, my theory on this when I was at *[facility name redacted],* another facility, is the cards don’t cost that much. I don’t care if I give you 20 of them in this year. Eventually, you’ll turn one in. Is our clinic getting charged for each card that we give out? I don’t think so, so if I give someone eight of them, I don’t care, as long as you eventually bring one of them back. Someone maybe could just give it as they check-in, even have it out in the hallway, some sort of prompting, so it’s not just the provider dependent.” (Tribe 1)	Not discussed

#### Increasing community demand

Both providers and community members placed considerable emphasis on one-on-one education in their respective focus groups. Providers spoke about underscoring CRC screening as a preventive measure (Table 3, P1) and the importance of using one-on-one interactions with patients as an opportunity for sharing information with them (Table 3, P2). Providers also noted the approach of using their own experiences of CRC screening as an example for patients, in an effort to personalize the process and make it less intimidating (Table 3, P3). Community members acknowledged interest in more one-on-one education to increase screening (Table 3, C1). They suggested providers and healthcare workers be mindful of the language that they use about cancer and cancer screening with community members in order to refrain from inducing fear and thereby dissuading interest in screening (Table 3, C2). Community members also highlighted the importance of trustful relationships between patients and physicians (Table 3, C3).

Community members and providers discussed patient reminders extensively as a promising strategy to encourage screening. Providers mentioned “reminder letters” and “reminder cards” to provide to community members to make them aware of their annual exam and any other tests they may be due for, including CRC screening (Table 3, P4-5). Providers suggested linking reminders to EHR to generate lists of patients on a regular basis who are due for screening. One suggestion was to identify birthdays of patients on a monthly basis and then send those patients a card to wish them a happy birthday accompanied with a reminder about their annual exam and/or screening (Table 3, P6). Community members repeatedly mentioned that hard copy reminders (letters, cards, etc.) delivered through the postal service would be more effective than text messages or phone calls (Table 3, C4-6).

Regarding small media, providers mentioned using newsletters to reach community members. They specifically noted that an existing newsletter provides a forum for fighters/survivors to write articles to share their experiences with readers (Table 3, P7). One provider also suggested playing videos of CRC screening “scenarios” in the waiting area of the healthcare facility to support education and awareness efforts (Table 3, P8). Community members likewise highlighted flyers (Table 3, C7), brochures (Table 3, C8), pamphlets (Table 3, C9), and mailed newsletters (Table 3, C10) as appropriate and effective small media for communicating CRC screening information. One participant cautioned against putting too much information on these materials as not to overwhelm community members; instead, the text should be “bold” font and provide “direct” information (Table 3, C10).

In one focus group, providers cited an existing client incentive program that facilitates monthly skill-building seminars for patients as a potential model for providing CRC screening information (Table 3, P9). The provider was optimistic about the incentive program’s potential to “make a difference,” noting that attendance for the existing incentive program continues to grow.

Regarding group education, providers said that it would be helpful to schedule regular (monthly or weekly) group education events about CRC screening, possibly in partnership with existing groups and other wellness initiatives in the community and healthcare facility (Table 3, P10). Providers highlighted the importance of group education to bring people together to share experiences about CRC screening so they “know that they’re not alone” (Table 3, P11). Likewise, community members acknowledged the utility of group education sessions and expressed interest in having more of them provided locally (Table 3, C11-13).

#### Increasing community access

Community members identified two key features of their experience at local tribally-operated healthcare facilities associated with efforts to reduce structural barriers to increase access to CRC screening. First, they noted the need for appropriate local (i.e., Tribal) language services (Table 3, C14-15). Second, community members expressed frustration with inefficiencies. This took the form of difficulty in scheduling appointments (Table 3, C16), lack of a “walk-in” option to see a provider (Table 3, C17-18), long wait times at the tribally-operated healthcare facility even with a scheduled appointment (Table 3, C19), perceived lack of urgency or seriousness by staff (Table 3, C20), and poor follow-up (Table 3, C21-22), in addition to high provider turnover and shortages.

#### Increasing provider delivery

Providers highlighted reminders as a potential strategy to engage patients about CRC screening in a regular and timely manner (Table 3, P12). Indeed, providers widely acknowledged that their EHRs have the functionality to generate automated provider reminders about client screening needs, including for CRC (Table 3, P13). However, providers repeatedly noted that there remain significant challenges to relying on such an approach. Broadly, their criticisms focused on limited time, staff, and resources to utilize and keep the EHR systems up to date regarding screening schedules for patients (Table 3, P13-18). Such limitations rendered the system inaccurate (Table 3, P14), “outdated” (Table 3, P15), vacant (Table 3, P16), misleading (Table 3, P17), and labor intensive (Table 3, P13-14, P16-18). For example, a provider shared that sometimes the tribally-operated healthcare facility can get very busy and they do not check the reminder box in the EHR to see whether the patient is due for a check-up (Table 3, P13).

Community members shared the belief that provider reminders would be helpful to encourage more CRC screening. They noted that screening information for individual patients should be available on patient charts (Table 3, C23-24) and on EHR systems (Table 3, C25) to prompt providers. Community members believed this would help with standardizing screening schedules (Table 3, C23) and being consistent with the screening recommendations for patients (Table 3, C24). Such systems documented providers’ efforts to remind patients, which may also serve to “protect” providers from criticisms of not engaging patients on the issue (Table 3, C25). Community members also suggested a “check sheet” for staff to complete for all new patients to ensure they are up to date on various exams and screenings (Table 3, C26).

Providers mentioned that assessments and feedback are an important strategy for supporting providers. However, there was some disagreement about how to share this feedback. There was a suggestion that it would be helpful to review facility-wide screening rates in comparison to their own in order to be aware of gaps and to make necessary improvements. It was also noted that provider-level data should remain private and not be available for other providers to review (Table 3, P19) to avoid singling out providers in the facility. There was some agreement with the value of reviewing facility-level data so long as provider-level data was left out altogether (Table 3, P20). One provider cited a negative experience with that kind of reporting at another facility as justification (Table 3, P20). Even though the idea of “multicomponent interventions” was not discussed extensively, many discussants suggested using several strategies simultaneously to improve CRC screening (Table 3, P21).

### Healthcare facility-level implementation and readiness

Tables 4 and 5 provide the results of the two surveys documenting the strategies being implemented at the two healthcare facilities and the ORIC measures, as reported by group-based responses from MAT team members. Overall, the implementation survey highlighted very few strategies being fully implemented at the healthcare facilities. In terms of readiness, the average scores for Tribe 1 were 3.3 and Tribe 2 were 4.2, indicating differing levels of readiness.

**Table 4:** MAT member survey on implementation from the two tribally-operated healthcare facilities

	Tribe 1 (*n* = 5)				Tribe 2 (*n* = 8)			
	Fully Implementing	Partially Implementing	Planning or open to implementing	Not implementing	Fully Implementing	Partially Implementing	Planning or open to implementing	Not implementing
**Increase community demand**								
• Education - Group education^a^			X				X	
• Education - One on one education^a^		X				X		
• Client reminder (text, email, mail, postcards)^a^			X				X	
• Mass media (TV, radio, Newspaper)				X				X
• Small media (brochures, flyers)^a^			X				X	
• Client incentives (cash, coupons)				X				X
**Increase community access**								
• Reduction of Out-of-Pocket Costs				X				X
• Reduction of Structural Barriers				X				X
**Increase provider delivery**								
• Provider reminder and recall systems^a^	X					X		
• Provider incentives			X				X	
• Provider assessment and feedback^a^			X				X	
**Other contextually relevant strategies**								
• Patient Navigation^a^			X				X	

^a^denotes EBI in survey

**Table 5:** MAT member survey on organizational readiness from the two tribally-operated healthcare facilities

	**Tribe 1 (*n* = 5)**	**Tribe 2 (*n* = 8)**
1. People who work here feel confident that the organization can get people invested in implementing this change.	4	5
2. People who work here are committed to implementing this change	3.5	4.5
3. People who work here feel confident that they can keep track of progress in implementing this change.	5	4
4. People who work here will do whatever it takes to implement this change.	3	4
5. People who work here feel confident that the organization can support people as they adjust to this change.	4	4
6. People who work here want to implement this change.	3	4.5
7. People who work here feel confident that they can keep the momentum going in implementing this change.	3	4
8. People who work here feel confident that they can handle the challenges that might arise in implementing this change.	4	4
9. People who work here are determined to implement this change.	3	4
10. People who work here feel confident that they can coordinate tasks so that implementation goes smoothly.	2	4.5
11. People who work here are motivated to implement this change.	3	4
12. People who work here feel confident that they can manage the politics of implementing this change.	2.5	4
**Average**	**3.3**	**4.2**

1 = Disagree; 2 = Somewhat Disagree; 3 = Neither Agree nor Disagree; 4 = Somewhat agree; 5 = Agree

### Implementation efforts at the two healthcare facilities

Findings from the focus group discussions and group-based assessments allowed the research team to engage with the MAT members in monthly meetings and collectively lead to the selection and implementation of interventions and strategies in the two healthcare facilities. This allowed the team to incorporate strategies to address the myriad gaps within each local healthcare facility that were identified as contributing to the historically low CRC screening rates among Tribal members. For example, both sites stressed the need to tailor and adapt system-level changes as a critical first step to ensure a coordinated, efficient, and sustainable approach to CRC screening promotion and delivery. The MAT at one healthcare facility identified nine interconnected system/community level changes, including: provider training, patient navigation, culturally appropriate small media, group education, patient reminders, EHR enhancements, provider assessment and feedback, FIT kit standing orders for nurses, and enhanced information exchange between the Tribal healthcare facilities and colonoscopy referral sites. The MAT at the second healthcare facility selected seven system- and community-level changes to incorporate including: provider training, patient navigation, culturally appropriate small media, group education, patient reminders, EHR enhancements, and community-healthcare facility linkages (i.e., FIT kit dissemination via mail and in community-based settings).

**Fig. 1:**
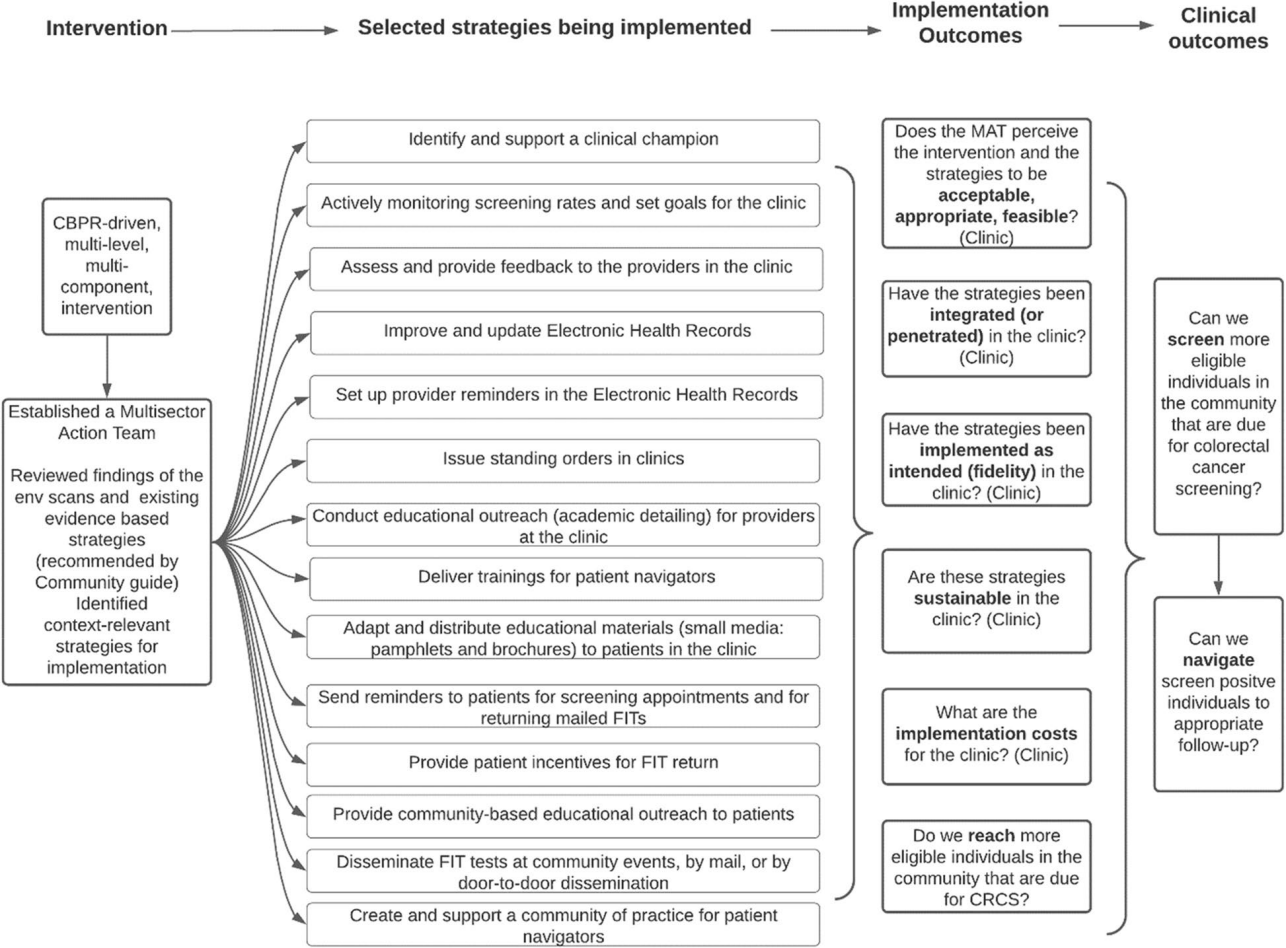
Interventions and strategies implemented for increasing the colorectal cancer screening rates at the healthcare facilities

Figure 1 shows the interventions and strategies that are being implemented by the two tribally-operated healthcare facilities. These selections were based in the data and engagement guided by the CBPR approach with the MAT members at the two tribally-operated healthcare facilities, who reviewed environmental scans and survey data on organizational implementation and readiness. Along with the facilitators, they collectively strategized and identified implementation strategies that were contextually-relevant for their healthcare facilities. The second column in Fig. 1 shows the specific strategies selected by the two MATs. These multilevel, multicomponent strategies are postulated to drive the Implementation and Clinical Outcomes (column 3 and 4) during the Implementation Phase of this study.

## Discussion

This formative research has provided information on how and under what conditions CRC screening processes can be implemented to improve uptake and reduce the CRC burden among AI communities served by the tribally-operated healthcare facilities in NM. The focus group discussions provided specific considerations in terms of support and feasibility from the perspectives of the Tribal community members and providers practicing at the healthcare facilities operated by these Tribes for the approaches outlined in The Community Guide [20]. With the organizational implementation and readiness data and the facilitation by the research team, the MATs planned and implemented strategies at their respective facilities. An iterative process, piloted strategies for their feasibility for implementation at each tribal healthcare facility to promote CRC screening, while targeting each level (i.e., individual, community, system). These strategies are being tested for the implementation and clinical outcomes in ongoing research with the communities.

It is important to note that the operationalization of many of these strategies within a tribally-operated healthcare facility required multiple steps. For instance, prior to implementation of an effective patient reminder system for CRC screening the following steps needed to be completed in each healthcare facility: (1) obtain and enter historical colonoscopies from external facilities, (2) create an EHR field to alert providers when a patient is due for CRC screening, (3) create an EHR function to track FIT kit dissemination (not just FIT completion), (4) create an EHR function to track completed reminders, (5) train providers on the utilization of new EHR functions, and (6) correct missing or inactive patient addresses and phone numbers. The process was similarly complex for Tribe 1 to operationalize provider assessment and feedback strategies. This included: (1) empanelling patients to a primary care provider, (2) determine who to empanel (i.e., define active patient population/denominator), (3) inform patients of their empanelment, (4) create an EHR function to track provider FIT kit dissemination, (5) train providers on the utilization of this new EHR function, and (6) create an EHR function to report CRC screening rates by provider.

The majority of these operational activities are now complete and the required components have either been implemented or are in the final stages of implementation. Both healthcare facilities have also engaged in extensive work to establish their baseline screening rates by entering all historical colonoscopies into their EHRs and defining their target population (i.e., AI, age 45–75, living in the Tribal community, at least two encounters at the healthcare facility in the past 3 years).

At the same time, we have maximized the readiness of the healthcare facilities at the two Tribes to fully implement and ultimately sustain their respective interventions in accordance with recommended national guidelines and strategies. As we move forward from the Planning and Pilot Phases to the Implementation Phase, the two Tribes and their healthcare facilities are well-situated to begin monitoring the efficacy of these novel interventions (i.e., changes in CRC screening rate), while continuing to place equal emphasis upon implementation indicators.

The study has some limitations that are being addressed in ongoing implementation efforts. The organizational assessments were conducted at one time point. With the extensive staff turnover in these facilities, data from these assessments may not be comprehensive or reflective of urrent perspective. In ongoing implementation efforts, our team has continued periodic discussions to note changes in these measures over the implementation period, recorded through detailed notes. Although conducting group-based assessments for the readiness of the tribally-operated healthcare facilities informed priorities in terms of interventions/strategies, for the research team it raised questions whether the presence of leadership influenced the selection of some strategies versus others. In the future, these assessments will be conducted individually and then discussed at a monthly MAT meeting. We believe, however, that findings from this community and clinical-partner engaged study provide a data-driven, nuanced understanding to implementation considerations that are relevant to the participating Tribes and their healthcare facilities. Using a facilitation-driven, participatory approach has informed the selection of contextually-relevant interventions and strategies in these resource-limited settings, which may contribute to effective implementation and sustainability of interventions and strategies in these settings.

## Conclusions

The findings from this study highlight the uniqueness of each Tribe in selecting and implementing specific strategies in its healthcare facility that collectively and synergistically contribute to the historically low CRC screening rates among Tribal members. A MAT was therefore essential to provide flexibility in addressing important cultural and contextual considerations and prioritizing strategies that would be implemented in each tribally-operated healthcare facility in accordance with recommendations from The Community Guide. Both tribally-operated healthcare facilities stressed the need to tailor and adapt system-level changes as a critical first step to ensure a coordinated, efficient, and sustainable approach to CRC screening delivery. We believe that guided by CBPR principles, such a model allows for additions of promising practices and adaptations that may be culturally appropriate and specific to each participating Tribe.

### Supplementary Information

Supplementary Material 1.

## Data Availability

The authors are committed to the open and timely dissemination of unique research outcomes in compliance with Tribal and federal (NIH) policies. The authors will share data being cognizant of the data sharing needs and goals of the participating Tribes, and are guided by the Tribal data sharing agreements.

## Abbreviations

ACCSIS: Accelerating Colorectal Cancer Screening and follow-up through Implementation Science
AASTEC: Albuquerque Area Southwest Tribal Epidemiology Center
AI/AN: American Indian and Alaska Native
CBPR: Community-Based Participatory Research
CPSTF: Community Preventive Services Task Force
CRC: Colorectal cancer
EBI: Evidence-based interventions
EHR: Electronic health records
GPRA: Government Performance and Results Act
IHS: Indian Health Service
IRB: Institutional Review Boards
I/T/U: IHS (I), Tribal (T) and Urban Indian (U)
MAT: Multisector action team
NCI: National Cancer Institute
NM: New Mexico
ORIC: Organizational Readiness for Implementing Change
UNM: University of New Mexico
UNMCCC: University of New Mexico Comprehensive Cancer Center
US: United States
USPSTF: United States Preventive Services Task Force
