# Supplementary material for: Participatory action research to develop and implement multicomponent, multilevel strategies for implementing colorectal cancer screening interventions in American Indian communities in New Mexico
Source: Implement Sci Commun. 2024 May 10;5:55. doi: 10.1186/s43058-024-00591-y (PMC11083750; doi:10.1186/s43058-024-00591-y)
Supplement: Supplementary file 1 — Supplementary Material 1. [file 43058_2024_591_MOESM1_ESM.docx]

# Supplementary materials

## Appendix 1. Recommendations for colorectal cancer screening from the Community Guide for Preventive Services

| **Intervention: FIT > colonoscopy**  **Implementation Strategy** | | | **Operational definitions** | **CPSTF recommendations** |
| --- | --- | --- | --- | --- |
| **Strategy** | **Type of Intervention** | **Intervention** |  |  |
| **Increase Community Demand** | Education | Group Education | Conveys info on indications for, benefits of, and ways to overcome barriers to CRC screening with the goal of informing, encouraging, and motivating participants to seek recommended screening; Can be given to a variety of groups, in different settings, and by different types of educators with different backgrounds and styles | Insufficient evidence |
|  |  | One-on-one education | Delivers info to individuals about indications for, benefits of, and ways to overcome barriers to CRC screening with the goal of informing, encouraging, and motivating them to seek recommended screening; Messages delivered by healthcare workers, lay health advisors, or volunteers, and are conducted by telephone or in person (medical, community, worksite, or household settings); Messages can be untailored to address the overall target population or tailored with the intent to reach one specific person | Recommended |
|  | Client Reminder | Text Messages, Email, Mail, Postcard | Written (letter, postcard, email); Telephone messages (including automated messages); Client reminders may be enhanced by: follow-up printed or telephone reminders; additional text/discussion with information about indications for, benefits of, and ways to overcome barriers to screening; assistance in scheduling appointments; Interventions can be untailored to address the overall target population or tailored with the intent to reach one specific person, based on characteristics unique to that person, related to the outcome of interest, and derived from an individual assessment | Recommended |
|  |  |  |  |  |
|  |  |  |  |  |
|  |  |  |  |  |
|  | Small Media | Video, Brochures, Letters, Newsletter, Pamphlets, Flyers, Social Media | Include videos and printed materials (letters, brochures, newsletters); Can be used to inform and motivate people to be screened; Can provide info tailored to specific individuals or targeted to general audiences | Recommended |
|  |  |  |  |  |
|  |  |  |  |  |
|  |  |  |  |  |
|  |  |  |  |  |
|  |  |  |  |  |
|  | Mass Media | Television, Radio, Newspaper, Magazines, Billboards | Includes TV, radio, newspapers, magazines, and billboards; Used to communicate educational and motivational information about CRC screening | Insufficient evidence |
|  |  |  |  |  |
|  |  |  |  |  |
|  |  |  |  |  |
|  |  |  |  |  |
|  | Client Incentives | Cash, Coupons | Small, non-coercive rewards (e.g., cash or coupons); Incentives are distinct from interventions designed to improve access to services (e.g., transportation, child care, reducing client out-of-pocket costs) | Insufficient evidence |
|  |  |  |  |  |
| **Increase Community Access** | Reduction of Out-of-Pocket Costs | Vouchers; Reimbursements; Reduction in copays; Adjustments in insurance coverage | Interventions attempt to minimize or remove economic barriers that make it difficult for clients to access CRC screening services; Costs can be reduced through a variety of approaches, including vouchers, reimbursements, reduction in co-pays, or adjustments in federal or state insurance coverage (MAILED FIT) | Insufficient evidence |
|  |  |  |  |  |
|  |  |  |  |  |
|  |  |  |  |  |
|  |  | Other |  |  |
|  | Reduction of Structural Barriers | Appointment Scheduling Assistance |  | N/A |
|  |  | Alternative Screening Sites/Hours |  | N/A |
|  |  | Transportation |  | N/A |
|  |  | Language Translation |  |  |
|  |  | Child Care |  |  |
|  |  | Patient Navigators | To deliver the FIT test, educate, and follow-up | Recommended |
|  |  | Limiting # of Clinic Visits |  |  |
|  |  | Other (mailed FIT) |  |  |
| **Increase Provider Delivery** | Provider Reminders and Recall Systems | EHR's | Reminders inform providers it's time for a client’s screening test; Recalls inform providers the client is overdue for screening; Can be provided in different ways, e.g. in client charts, via e-mail | Recommended |
|  |  | Email |  |  |
|  |  | Patient Chart |  |  |
|  |  | Other |  |  |
|  | Provider Incentives | Monetary Rewards; Continuing Education Credit | Direct or indirect rewards intended to motivate providers to perform screening or make appropriate referral for their patients to receive these services; Rewards are often monetary, but can also include nonmonetary incentives (e.g., continuing medical education credit) | Insufficient evidence |
|  |  |  |  |  |
|  |  | Other |  |  |
|  | Provider Assessment and Feedback | Provider Assessment and Feedback | Both evaluate provider performance in delivering/offering screening to clients (assessment) and present providers with info about their performance in providing screening services (feedback); Feedback may describe the performance of a group of providers (e.g., mean performance for a practice) or an individual provider, and may be compared with a goal or standard | Recommended |
|  |  |  |  |  |

## Appendix 2: Qualitative Interview Guide - Leadership and providers

Practice Name: _____________________________________________

Location and Address: ________________________________________

Leadership type: ______________________________________

How long have you worked in this clinic/system: _____________________

IHS clinic ________________________urban clinic____________________

Tribal clinic (638) ________________Other (e.g., FQHC) ________________________

Participant ID: ________

Thank you so much for taking the time to talk with me today. The purpose of this study is to develop a program to increase colorectal cancer screening among Native American communities across Arizona, New Mexico, and Oklahoma. Your clinic has joined this program (or is considering joining this program). As a stakeholder in this practice, we would like to know your perspective on colorectal cancer screening efforts in your clinic (or health care facility). Please feel free to elaborate on your responses as much as needed. Your participation in this interview (or focus group) is completely voluntary. You can refuse to answer any of the questions and you can stop participating in the interview (or focus group) at any time. The interview (or focus group) is anonymous and we will keep all collected information confidential.

**CONFIDENTIALITY**

- To accurately interpret and analyze the data from this interview (or focus group), I will be taking some notes during the discussion. As explained in the consent, I will also be audio recording the interview to make sure that our notes are accurate and that we do not miss any important parts of the discussion.
- Although I will be recording the session, any and all responses and comments that are made during the interview (or focus group) will remain confidential.
- Only authorized research team members will have access to the data from this interview (or focus group), which will be stored on a password protected computer and in a locked cabinet in a secured office at AAIHB and UNM.
- The audio tapes from the session will be destroyed as soon as the information from them is transcribed and verified.
- The transcriptions will not contain any names or personal identifiers that can be linked to you.

**HOW WILL THE INFORMATION BE USED AND REPORTED?**

- We will prepare a report based on the interviews (or focus groups). We will share this report with our Community Advisory Committee and Program Steering Committee, and may also disseminate it through academic publications.
- The final report may contain some individual quotations from the interviews (or focus groups); however, specific names will not be included in the report.
- The ultimate goal is to develop a colorectal cancer screening program in the communities that is effective and culturally sensitive.

[Note to facilitator: In addition to the questions provided below, you are encouraged to ask clarifying questions in response to respondent’s answers. The questions do not need to be asked in sequential order in cases where the respondent addresses a particular topic while answering another question.]

**Screening Practices and Referral Procedures:**

- What specific tests for colorectal cancer screening are used in your health care facility (or health care system)?
- Are there additional screening practices that could be used, and what kinds of considerations would impact these decisions? (provide a reference table of colorectal cancer screening strategies from United States Preventive Services Task Force for review).
- Does this health care facility (or health care system) currently have a multisector coalition or individual champion that leads efforts to enhance colorectal cancer screening?
  - If yes, who? What sectors are represented?
- What is the process whereby a patient at average risk is referred and screened for colorectal cancer? (Prompts: who would they see or what do they do next; who would follow up with the patient? Who would follow-up if they patient did not complete the test? How long does follow up typically take? How far away are testing locations?)
- What are the procedures for follow-up of positive colorectal cancer screening results? (Prompts: who would they see or what do they do next; who would follow up with the patient? Who would follow-up if the patient did not receive or access diagnostic test? How long does follow up typically take? How far away are testing locations?)
- What, if anything, would you do to improve the process of colorectal cancer screening at this health care facility (or health care system)?
- What specific strategies can be used to improve communication with patients about the importance of colorectal cancer screening?
- What are some of the challenges or successful strategies for engaging the community about colorectal cancer screening?

**System-wide questions:**

- How does colorectal cancer screening fit into the overall priorities for this health care facility (or health care system)?
- How are you trying to align colorectal cancer screening with the many other aspects of patient care (e.g., other screening tests, chronic disease management, community outreach, etc.)
- Can you describe what is being done throughout this health care facility (or health care system) as it relates to colorectal cancer screening? Probe – guidelines used (ACS, USPSTF, other)
- What systems are currently in place to ensure that all average risk patients are recommended for CRC screening? Probes: policies, provider reminder/recall systems, provider assessment and feedback, provider incentives, patient reminders, patient incentives, navigators, small media, EHR prompts, one on one education, group education, reduction in administrative barriers, assisting in scheduling appointment, use alternate screening hours, provide transportation, provide child care, use alternate screening sites, other approaches
- Can you talk about any specific challenges or needs as it relates to colorectal cancer screening practices at this health care facility (or health care system)? Probes – resources, insurance, patient specific barriers (e.g. fear, stigma, fatalism, taboos), manpower of facility, etc.
- Can you identify specific strengths as it relates to colorectal cancer screening practices at this health care facility (or health care system)? What kinds of things are working well?
- To what do you attribute some of the successes related to colorectal cancer screening efforts in this health system?
- Are there any other areas that need improvement as it relates to colorectal cancer screening at this health care facility (or health care system)?
- Have any cultural considerations of community members been taken into account in the establishment of your current colorectal cancer screening system/activities?
- Is there anything you would add to this…something I have not asked about?

Thank you so much for your time. We will combine all of the responses from various stakeholders to develop themes to inform our program

**Qualitative Interview Guide – Community Stakeholder**

Current clinic: _____________________________________________

Location and Address: ________________________________________

Primary clinic where you receive care: ____________________________

How long has that been your primary clinic: ________________________

Role (Patient, not a current patient, other): ______________________________________

IHS facility: Yes No

If no, other type: (eg., 638; FQHC, etc): ___________________________

Thank you so much for taking the time to talk with me today. The purpose of this study is to develop a program to increase colorectal cancer screening among Native American communities across Arizona, New Mexico, and Oklahoma. As a stakeholder engaged in improving the health and well-being in Native American communities, we would like to know your perspectives on colorectal cancer screening in your community.

Please feel free to elaborate on your responses as much as needed.

Your participation in this interview (or focus group) is completely voluntary. You can refuse to answer any of the questions and you can stop participating in the interview (or focus group) at any time. The interview (or focus group) is anonymous and we will keep all collected information confidential.

**CONFIDENTIALITY**

- To accurately interpret and analyze the data from this interview (or focus group), I will be taking some notes during the discussion. As explained in the consent, I will also be audio recording the interview to make sure that our notes are accurate and that we do not miss any important parts of the discussion.
- Although I will be recording the session, any and all responses and comments that are made during the interview (or focus group) will remain confidential.
- Only authorized research team members will have access to the data from this interview (or focus group), which will be stored on a password protected computer and in a locked cabinet in a secured office at AAIHB and UNM.
- The audio tapes from the session will be destroyed as soon as the information from them is transcribed and verified.
- The transcriptions will not contain any names or personal identifiers that can be linked to you.

**HOW WILL THE INFORMATION BE USED AND REPORTED?**

- We will prepare a report based on the interviews (or focus groups). We will share this report with our Community Advisory Committee and Program Steering Committee, and may also disseminate it through academic publications.
- The final report may contain some individual quotations from the interviews (or focus groups); however, specific names will not be included in the report.
- The ultimate goal is to develop a colorectal cancer screening program in the communities that is effective and culturally sensitive.
- [Note to facilitator: In addition to the questions provided below, you are encouraged to ask clarifying questions in response to respondent’s answers. The questions do not need to be asked in sequential order in cases where the respondent addresses a particular topic while answering another question.]

**Personal Experiences:**

- First, we’re interested in knowing what health issues are most important to people here?

Prompt: What about cancer—is that something that people are concerned about?

- Now we’d like to talk more about colorectal cancer. Has this cancer been an issue here in the community? Prompts: Are people worried about colorectal cancer? What have you heard about being screened for colorectal cancer? from whom? (PROBE)

Moderator: Just to make sure we are all on the same page about the term “screening,” I’d like to give a brief definition. Screening is a word or term used for getting tested for cancer. This does not mean you have cancer or that your provider suspects that you have cancer. As we get older, medical guidelines recommend that everyone gets checked or tested for different types of cancer and it is called screening

- Thinking about these kinds of screening tests, would anyone like to share a personal experience with colorectal cancer screening? what type of test was it? What was your experience with the screening test? (PROBE: accessing materials or site; understanding instructions) Was any type of follow-up testing recommended? If yes, what was your experience in completing the follow-up testing.
- We understand that many people haven’t done a screening test for colorectal cancer and there can be many reasons for this. Can someone who hasn’t been tested share any of these reasons? Prompts: Some challenges that people have mentioned include things like: they didn’t know about the test, concerns about how much it might cost, it’s hard to follow the test instructions, etc…
- Regardless of whether you’ve done of these tests, we’d now like to hear about whether your provider has ever asked you to consider getting screened for colorectal cancer? If so, what screening test(s) was recommended? If not, how would you feel if your provider asked you to get screened for colorectal cancer? What kinds of information about the test is important for your provider to share with you?
- Now that you’ve been thinking about this colorectal cancer screening test, how much of a priority is it for you? For members of your family? Members of your community?
- Do you think people in the community are getting screened for colorectal cancer? Why or why not?
- Can you think of some ways to increase the number of people getting colorectal cancer screening?
- What do you think are some specific challenges in reaching out to the community about
- colorectal cancer? What would make it easier for people to get screened?
- What (if any) cultural considerations should be taken into account by the clinic related to colorectal cancer screening among community members?

**Education/Outreach**

- For those of you who have heard about colorectal cancer screening, where do you get your information from?
- Do you talk to anyone about colorectal cancer? If so, who (what is their relationship to you)?
- How would you like to hear about colorectal cancer screening? How do you think your family would like to hear about it?
- Do you have reliable access to the Internet? Do you seek health care information online? Why or why not?
- Do you think you would you use a mobile application to find health care information?
- Is there anything you would add to this…something I have not asked about?

Thank you so much for your time. We will combine all of the responses from various stakeholders to develop themes to inform our program.
